# Therapeutic mechanisms of genistein in ischemic stroke: A systematic review of *in vivo* and *in vitro* studies

**DOI:** 10.1371/journal.pone.0338590

**Published:** 2025-12-23

**Authors:** Nurin Yasmin Mohd Khairudin, Awla Mohd Azraai, Rosfaiizah Siran, Nasibah Azme

**Affiliations:** 1 Institute of Medical Molecular Biotechnology, Universiti Teknologi MARA (UiTM), Sungai Buloh, Selangor, Malaysia; 2 Laboratory Animal Care Unit, Universiti Teknologi MARA (UiTM), Sungai Buloh, Selangor, Malaysia; 3 Department of Pathology, Faculty of Medicine, Universiti Teknologi MARA (UiTM), Sungai Buloh, Selangor, Malaysia; 4 Multidisciplinary Initiative in Neuroscience Discovery and Science (MINDS), Faculty of Medicine, Universiti Teknologi MARA (UiTM), Sungai Buloh, Selangor, Malaysia; 5 Department of Physiology, Faculty of Medicine, Universiti Teknologi MARA (UiTM), Sungai Buloh, Selangor, Malaysia; 6 Department of Medical Education, Faculty of Medicine, Universiti Teknologi MARA (UiTM), Sungai Buloh, Selangor, Malaysia; Helwan University, EGYPT

## Abstract

Genistein is an isoflavone phytoestrogen that is considered a nutraceutical compound found in soybean. The mimicking of estrogen effects includes the ability to bind to the intracellular and cell membrane receptors of estrogen and exert biological functions like antitumor, anti-inflammatory, anti-oxidative, and antiproliferative properties. With more studies focusing on the therapeutic effect of genistein, both *in vitro* and *in vivo*, it is evident that genistein acts through multiple pathways including anti-apoptotic, anti-inflammatory, and anti-oxidative. As the effects of stroke are affecting more people and causing devastating repercussions, this warrants genistein to be utilized as a therapeutic drug. Therefore, further studies are due on the effects of genistein on humans so that clinical trials can be carried out for long-term benefits. This review encompasses various studies regarding the potential neuroprotective effects of genistein on cerebral stroke, examining both *in vitro* and *in vivo* experimental models. Four database searches: Web of Science (WoS), Scopus, PUBMED and Science Direct were searched from 1^st^ January 1999 until 31st October 2025. The initial datasets identified through the database search yielded a total of 549 publications and 341 publications were finalized after removing duplicates. In the initial screening, a total of 293 studies were excluded due to their irrelevance to the main objective of this study. After assessing the suitability of the studies and following the PRISMA guidelines, a total of 31 articles were found to be suitable and systematically reviewed. Findings demonstrated the major mechanistic pathways involved in the therapeutic action of genistein are anti-apoptotic, anti-inflammatory, and anti-oxidative. Each of these mechanisms is governed by specific pathways, which will be thoroughly discussed, indicating that genistein can be effective as a therapeutic drug in ischemic stroke.

## 1. Introduction

Cerebral stroke is a complex medical condition caused by decreased blood flow to the brain and is a major cause of disability and mortality in both developing and developed countries [1]. The main cause of stroke is high blood pressure where other factors such as smoking,diabetes,obesity.The symptoms varies according to different person.It includes severe headache,weakness in limbs,slurred speech,vision problems and paralysis [2]. Almost 80% of reported stroke cases are Ischemic strokes which is caused by thrombosis, embolism, and/or hypoperfusion [3]. Pathological events contributing to the development of ischemic stroke include neuroinflammation, which plays a significant role in the acute cerebral ischemia/ reperfusion injury (I/RI) cascade [4]. As neuroinflammation triggers a massive immune response in ischemia, the overstimulated production of microglia that leads to the activation of an inflammatory cascade causes irreversible necrotic neuronal death [4].

Currently,Tissue plasminogen activator is the only FDA-approved and widely accepted treatment for ischemic stroke which is considered as the gold standard in medical field [4,5]. However due to its limitations and associated risk it is important to investigate about any potential medication that could serve as an intervention and prevention for stroke. The compound widely discussed for treating Ischemic stroke is Genistein Researchers are intrigued with the fact of using phytoestrogen for the neuroprotective treatment of stroke. Genistein is a nutraceutical compound that can be found in soybean, clover, Puerarin, horn, dyewood, and broad bean root. It is an isoflavone phytoestrogen that constitutes approximately half of the total isoflavones found in soy food and is structurally similar to estrogen which also mimics estrogen’s effects [4,6]. This mimicking includes binding to both intracellular and cell membrane estrogen receptors, enabling it to perform various biological functions including antitumor, anti-inflammatory, anti-oxidative, and antiproliferation properties [4]. The addition of genistein in dietary ingestion may offer varied health benefits, including chemoprevention of certain types of cancer, cardiovascular disease, and post-menopausal ailments [6]. In the context of ischemic stroke, previous research has found that genistein has neuroprotective properties against cerebral ischemia/reperfusion injury(I/RI). These effects are mediated through mechanistic pathways including deactivation of signal transducer and activator of transcription 3 (STAT3) deactivation, alpha-7 nicotinic acetylcholine receptor (α7nAChR) and Nuclear factor kappa B (NF-κB) (α7nAChR-NF-κB) signaling pathway,regulation of phosphatidylinositol-3-kinase (PI3K)/protein kinase B (Akt)/mammalian target of rapamycin (mTOR) (PI3K-Akt-mTOR) pathway, up-regulation of nuclear factor-erythroid factor 2-related factor 2 (Nrf2), c-Jun N-terminal kinase (JNK) and extracellular signal-regulated kinase (ERK) signaling pathway, and inhibition of nucleotide-binding domain, leucine-rich–containing family, pyrin domain–containing-3 (NLRP3) inhibition [1,3,4,7–9]. This systematic review aims to evaluate the therapeutic mechanisms of genistein in ischemic stroke, based on findings from both *in vivo* and *in vitro* studies. The review will follow the PICO framework, focusing on the population affected (ischemic stroke models), the intervention (genistein), and the outcomes related to key mechanistic pathways.

## 2. Methodology

This review was done according to the reporting standards suggested in the Preferred Reporting Items for Systematic Reviews and Meta-Analyses (PRISMA) statement [10]. The protocol for this systematic review was registered with INPLASY (International Platform of Registered Systematic Review and Meta-analysis Protocols; with unique ID number 6749) and is available in full on inplasy.com (https://doi.org/10.37766/inplasy2024.9.0010).

### 2.1. Comprehensive literature review

A systematic search was conducted using bibliographic databases and other evidence sources which addressed the search question. The comprehensive literature search involved looking at the eligibility of articles, searching strategies for identification of studies, study selection, and data extraction.

### 2.2. Formulating search question

The search was performed by identifying the type of evidence needed to answer the search question. A strategy of using the Domain, Determinant, and Outcome (DDO) format was used in the study to obtain relevant answers to the formulated question. The strategy was as follows:

Domain – “Stroke” OR “Cerebral Ischemia” OR “Brain Ischemia” OR “Ischemic Stroke
Determinant – “Genistein” OR “Soy Isoflavones”
Outcome – “Mechanism”

Thus the formulating search question is,

How does genistein display its mechanism in the treatment of ischemic stroke, as demonstrated in *in vivo* and *in vitro* studies?

### 2.3. Eligibility criteria

The inclusion and exclusion criteria for this study is presented and summarized in Table 1.

**Table 1:** Eligibility criteria for this study.

Criteria Description	Inclusion Criteria	Exclusion Criteria
Study Type	• *In vivo* (animal models) or *in vitro* (cell cultures) investigating genistein in ischemic stroke	• Clinical trials or studies involving human subjects
Population	• Animal models of ischemic stroke (for *in vivo* studies) • Cell cultures subjected to ischemic-like conditions (for *in vitro* studies)	• Studies that do not focus on ischemic stroke models
Intervention	• Studies administering genistein or soy isoflavones as an intervention	• Studies that do not include genistein or soy isoflavones as an intervention
Mechanistic Focus	• Studies investigating mechanisms related to genistein’s therapeutic effects	• Studies focusing purely on clinical or behavioral outcomes, without exploring mechanisms
Outcome	• Studies reporting mechanistic outcomes such as cellular pathways	• Studies that report only clinical outcomes without mechanistic insights
Language	• English medium	• Studies published in languages other than English
Publication Type	• Original article as the source of this review	• Review articles, book chapters, meeting abstracts/conference proceedings

### 2.4. Search strategy for identification of studies

The identification of studies included all published studies. The Boolean search was performed on each database using the search terms: (“genistein” OR “soy isoflavone”) AND (“stroke” OR “cerebral ischemia” OR “brain ischemia” OR “ischemic stroke). The published literature was thoroughly searched. The database was searched for articles published between January 1, 1999, and October 31, 2025. This review includes the research papers from 1999 onward focusing on genestein’s therapeutic potential in the treatment of Ischemic stroke particularly in *in vivo* and invitro studies.The period starting from 1999 was chosen because it had significant advancements in understanding genistein’s mechanism of action and target pathways. Hence by focusing on studies from this time frame, the review aims to include more accurate research findings related to genistein and its relevance with ischemic stroke.

### 2.5. Identification of published articles

Published articles refer to any article that has been published in any publishing journal platform. The first step was to locate all relevant published articles for this research using a computer-based information search. The established databases in this study were Web of Science (WoS), Scopus, PubMed, and Science Direct. The references of the chosen studies were then analyzed manually by all researchers.

### 2.6. Screening and data extraction

Screening of titles and abstracts was conducted independently by two reviewers (N.Y.M.K. and N.A.). Disagreements were resolved through discussion with two other reviewers (R.S. and A.M.A.). Full-text articles were then retrieved, and eligible studies were uploaded into EndNote for organization. Data extraction was performed by the same two reviewers to ensure inter-rater reliability and minimize data entry errors. The extracted data were organized and tabulated in Microsoft Excel.

### 2.7. Full text retrieval

Full-text articles of eligible studies were obtained and downloaded from Web of Science (WoS), Scopus, PUBMED and ScienceDirect. Only articles that offer free access were included in this study. Articles without full text were excluded from this study.

### 2.8. Selection and inclusion of final articles

The selection of final full-text articles was based on predefined eligibility criteria. Disagreements were discussed until consensus was reached among the four reviewers. The finalized articles were manually coded and analyzed using structured data extraction forms and Excel.

### 2.9. Critical appraisal

A process of critical appraisal was performed by N.Y.M.K and N.A to assess the article quality and appropriateness of study design to the research objective. The critical appraisal of the selected articles was performed by using the modified version of the “McMaster University’s Critical Review Form” [11] to extensively analyse and evaluate the articles in following domains as listed in the review form: study purpose, literature, study design, appropriateness of study design, outcomes, results, and conclusion of studies [12]. Excel files were prepared based on key assessment report checklists to make the appraisal process easier. The researchers conducted critical assessments in duplicate and if there was disagreement, consensus will be reached with other researchers regarding the credibility of the articles.

### 3.0. Flowchart of review process

**Fig 1:**
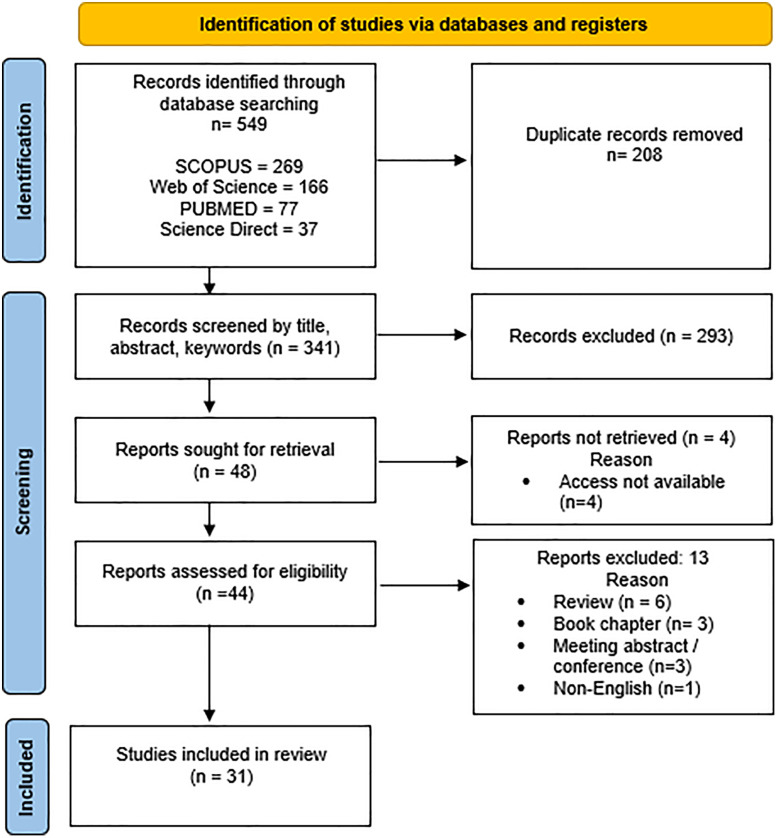
Flowchart of the review process.

The overall process of article identification, screening, eligibility assessment, and final inclusion is illustrated in Fig 1.

## 3. Results

The process of searching and selecting articles performed in this study is summarized in Fig 1. A total of 549 records were initially identified through database searches. After removing duplicates, 341 articles remained. Following the first screening phase, 293 studies were excluded due to irrelevance to the study objective. Ultimately, 31 articles met the eligibility criteria and were included in this systematic review.

All selected studies investigated the therapeutic potential of genistein or its derivative, genistein-3-sodium sulfonate (GSS), in ischemic stroke models. However, the experimental designs varied. This review found that of 30 studies, 24 studies employed *in vivo* models using animal subject, 5 studies used *in vitro* models involving cell cultures, and 2 studies used both *in vivo* and *in vitro* approaches. The findings from all 31 studies are compiled in Table 2, which includes study models, doses, duration, routes of administration, induced conditions, targeted pathways, mechanisms of action and outcomes.

**Table 2:** Summary of included studies investigating the therapeutic mechanisms of genistein in ischemic stroke.

StudyNo	Author, Year	Model	Genistein Dose/ Concentration	Duration	Route of Administration (For *In vivo* Studies)	Stress/exposure (Induced condition/ experimental insult)	Pathway(s) involved	Mechanism of Action for Genistein	Outcomes/Endpoints	Reference
1	Trieu, Vuong N. and Fatih M. Uckun 1999.	Murine model of familial ALS and stroke	16 mg/kg, twice daily	24 hours prior to irradiation until 24 hours after irradiation	Intraperitoneal injection	Familial ALS (G93A SOD1 transgenic mice) and free radical-induced cerebral ischemia (stroke model via photochemical damage)	Estrogen receptor-dependent and independent mechanisms, Tyrosine kinase inhibition, Inhibition of ROS (Reactive Oxygen Species)	In ALS model: Genistein delayed disease onset in males by acting as a phytoestrogen through estrogen receptor signaling, reducing hydroxyl radical formation caused by SOD1 mutation. In stroke model: Genistein acted through tyrosine kinase inhibition, reducing ROS and oxidative damage, blocking the apoptotic pathway triggered by free radicals.	Delayed ALS onset in males, reduced mortality; smaller infarct size in stroke model, reduced lesion volume	[13]
2	Li, Hong-Chun; Zhang, Guang-Yi, 2003	Rat transient global cerebral ischemia (hippocampus, 4-vessel occlusion; 15 min ischemia, up to 72 h reperfusion)	5, 10, 15, 20 mg/kg	Single dose 20 min before ischemia; readouts at 72 h	Intraperitoneal injection	Brain ischemia/reperfusion (4-vessel occlusion)	JAK/STAT3 signaling; PTK-dependent STAT3 Tyr705 phosphorylation & nuclear translocation	Genistein dose-dependently inhibits STAT3 tyrosine phosphorylation (cytosolic & nuclear) and STAT3 DNA-binding activity; total STAT3 unchanged	mechanistic endpoints only (no infarct/behavior)	[14]
3	Liang, Hua-Wei, Shui-Feng Qiu, Jia Shen, Li-Na Sun, Jing-Ye Wang, Iain C. Bruce, and Qiang Xia. 2008.	Rat model of transient global cerebral ischemia (hippocampal CA1 region)	15 mg/kg	One injection 30 min before and again at 24 h after ischemia	Intraperitoneal injection	Transient global cerebral ischemia (induced by 4-vessel occlusion for 10 min)	Mitochondria-mediated apoptotic pathway, ROS inhibition, NMDA receptor modulation	Attenuates mitochondrial ROS production, decreases lipid peroxidation (malondialdehyde (MDA)), inhibits cytochrome c release, and prevents caspase-3 activation. Inhibits tyrosine kinase activity, reduces glutamate release, and blocks Ca2 + influx through NMDA receptor modulation	Reduced neuronal damage, decreased TUNEL-positive apoptotic cells, reduced ROS, decreased cytochrome c release, inhibition of caspase-3 activation, and decreased lipid peroxidation (MDA)	[15]
4	Schreihofer, Derek A. and Lori Redmond, 2009	Primary embryonic rat cortical neurons (*in vitro*)	0.1–1 µM	24 hours pretreatment before insult	N/A *In vitro* (cell culture)	Hypoxia, oxygen-glucose deprivation (OGD) for 2h or 5h, glutamate toxicity, thapsigargin-induced apoptosis	Estrogen receptors (ERs), phosphoinositide 3-kinase (PI3K), mitogen-activated protein kinase (MAPK), MAPK/ERK (extracellular signal-regulated kinase)	Inhibition of apoptosis and caspase activation via ERs; neuroprotection mediated by PI3K and ERK pathways	Reduced cell death and LDH release, inhibition of apoptosis, enhanced cell survival during hypoxia and OGD	[16]
5	Ma, Yulin, Jennifer C. Sullivan, and Derek A. Schreihofer, 2010	Male and OVX (ovariectomized) female Sprague-Dawley rats subjected to transient middle cerebral artery occlusion (tMCAO)	500 ppm (Genistein) in diet	2 weeks prior to tMCAO followed by 72 hours of reperfusion	p.o. (Dietary)	Transient focal cerebral ischemia (90 minutes occlusion of the middle cerebral artery)	NADPH oxidase (NOX) inhibition, superoxide reduction, Estrogen Receptor (ER) activation	Decreases NOX activity and superoxide production, reduces oxidative stress via ER-mediated mechanisms, and upregulates NQO1 and ERCC2 genes, involved in antioxidant defense and DNA repair	Reduced infarct size, decreased oxidative stress markers (superoxide, TBARS), improved neurological outcomes	[17]
6	Castello-Ruiz M, Torregrosa G, Burguete M, Salom J, Gil J, Miranda F… et al. 2011	Wistar, WKY, and SHR rat models of ischemic stroke	1 mg/kg/day (Genistein)	3 days post-ischemia (90 min occlusion of MCA followed by reperfusion)	Intraperitoneal injection	Transient focal cerebral ischemia (MCA occlusion for 90 minutes followed by 3 days of reperfusion)	Estrogen receptors (ERα, ERβ), cerebral circulation regulation	Genistein acts through estrogen receptor modulation, improving cerebral blood flow and reducing infarct volume by relaxing cerebral arteries and enhancing microcirculation. No effect in hypertensive (SHR) and WKY rats.	Significant reduction in infarct volume in Wistar rats (50% reduction). No effect in WKY or SHR rats. Improved cerebral circulation in Wistar rats.	[18]
7	Qian, Yisong, Liangxun Cao, Teng Guan, Lan Chen, Hongbo Xin, Yunman Li, Rui Zheng, and Deyue Yu, 2012	Male C57/BL6J mice	2.5, 5, and 10 mg/kg, once daily	14 days pretreatment followed by 24 hours of reperfusion	p.o. (intragastric administration)	Transient focal cerebral ischemia (1-hour occlusion of MCA followed by 24 hours reperfusion)	Mitochondria-dependent apoptotic pathway, ROS-NF-κB signaling	Genistein suppresses ROS production in mitochondria, inhibits cytochrome c release and caspase-3 activation, preventing apoptosis. It also inhibits NF-κB activation, downregulating phosphorylated IκB and NF-κB p65, disrupting the ROS-NF-κB feedback loop.	Reduced infarct size, decreased apoptosis (TUNEL assay), improved antioxidant enzyme activity (SOD, GPx), and reduced oxidative stress (MDA levels, ROS production)	[6]
8	Cortina, Belén, Germán Torregrosa, María Castelló-Ruiz, María C. Burguete, Antonio Moscardó, Ana Latorre, Juan B. Salom, Juana Vallés, María T. Santos, and Enrique Alborch, 2013	Wistar Rat model of Middle Cerebral Artery Occlusion (MCAO)	10 mg/kg/day	3 days	Intraperitoneal	Ischemia-reperfusion (Middle cerebral artery occlusion, MCAO)	Thromboxane A2, Leukocyte-platelet Aggregates, Ca2 + Influx	Genistein improved circulatory function by reducing thromboxane A2 production, lowering leukocyte–platelet aggregates and restoring carotid artery reactivity.	Significant reduction in thromboxane A2 concentration, lower leukocyte-platelet aggregates, restored carotid artery contractile responses. Improved functional scores and reduced infarct size post-stroke.	[5]
9	Wang, Ruimin, Jingyi Tu, Quanguang Zhang, Xi Zhang, Ying Zhu, Wendong Ma, Cheng Cheng, Darrell W. Brann, and Fang Yang, 2013	Adult male Sprague Dawley rat model of GCI	1 mg/kg	Administered 5 minutes post-reperfusion	Intravenous (i.v.) injection	Global cerebral ischemia (4-vessel occlusion)	eNOS/Nrf2/HO-1 pathway, S-nitrosylation of Keap1	Genistein activates eNOS to increase NO production, which promotes the S-nitrosylation of Keap1. This dissociates Nrf2 from Keap1, allowing Nrf2 to translocate to the nucleus and upregulate the HO-1 antioxidant response. Genistein’s neuroprotection is mediated through reduction of oxidative stress and inhibition of apoptosis.	Reduced oxidative stress (8-OHdG and 4-HNE markers), decreased apoptosis (TUNEL assay), improved spatial memory and cognitive function, increased survival of hippocampal neurons (NeuN-positive cells)	[19]
10	Shambayati, Maryam, Maharshi Patel, Yulin Ma, Rebecca L. Cunningham, and Derek A. Schreihofer, 2014	Ovariectomized Sprague-Dawley rat model of tMCAO	Teklad diet 600 μg/g soy isoflavones and results in an average of 6 μM circulating total isoflavones	5 weeks pretreatment, followed by ischemic insult	p.o. (Dietary)	Transient middle cerebral artery occlusion (tMCAO) for 90 minutes followed by reperfusion	NF-κB inhibition, cytokine suppression (IL-1β, IL-2, IL-13, CXCL1), microglial modulation	Genistein-rich soy diet inhibits NF-κB translocation, leading to reduced production of pro-inflammatory cytokines (IL-1β, IL-2, IL-13, CXCL1). It also reduces the activation of microglia, preventing further damage to brain tissue.	Reduced inflammatory response, including decreased levels of IL-1β, IL-2, IL-13, CXCL1. Decreased activation of microglia in the infarct core.	[20]
11	Shi, Rengfei, Shunli Wang, Xiang Qi, Si Chen, Peijie Chen, and Quanguang Zhang, 2014	Ovariectomized female Sprague-Dawley rat model of forebrain ischemia	0.1 mg/kg/day via osmotic minipumps	14 days pretreatment followed by ischemic insult	Subcutaneous osmotic pumps	Transient forebrain ischemia (4-vessel occlusion model)	Glucocorticoid receptor (GR) signaling, Mdm2-ubiquitination pathway	Genistein inhibits GR nuclear translocation and DNA-binding activity, reduces GR levels via Mdm2-targeted proteasomal degradation, and prevents GR signaling-induced neuronal damage and microglial activation.	Reduced GR levels in hippocampal CA1 region, decreased microglial activation, increased neuronal survival in CA1 pyramidal cells, reduced apoptosis.	[21]
12	Wang, Shiquan, Haidong Wei, Min Cai, Yan Lu, Wugang Hou, Qianzi Yang, Hailong Dong, and Lize Xiong, 2014	Ovariectomized mice with transient cerebral ischemia (MCAO model)	10 mg/kg	14 days prior to ischemic insult	Intraperitoneal injection	Transient middle cerebral artery occlusion (MCAO)	ERK1/2 signaling pathway	Genistein activates ERK1/2 phosphorylation, promoting neuronal survival through the upregulation of Bcl-2 and downregulation of Bax, leading to reduced apoptosis. ERK inhibition blocks genistein’s protective effects, confirming its dependence on the ERK pathway.	Reduced infarct size, improved neurological outcomes, decreased apoptosis (TUNEL-positive cells), increased Bcl-2/Bax ratio.	[22]
13	Aras, Adem Bozkurt, Mustafa Guven, Tarik Akman, Hasan Alacam, Yildiray Kalkan, Coskun Silan, and Murat Cosar, 2015	Male Sprague-Dawley rat model of MCAO	10 mg/kg	Administered at 5th minute after ischemia	Intraperitoneal injection	Transient focal cerebral ischemia via MCAO	Oxidative stress reduction, Mitochondria-mediated apoptotic pathway, NRF1 activation	Genistein increases SOD and NRF1 levels, reduces MDA (lipid peroxidation marker), and inhibits apoptosis by reducing caspase-3 and caspase-9 activity. Genistein mitigates neuronal degeneration and reduces oxidative stress and apoptosis through mitochondrial protection.	Reduced oxidative stress, increased SOD and NRF1 activity, decreased MDA levels, and reduced caspase-3 and caspase-9 activity. Improvement in neurological deficits and reduced neuronal damage.	[23]
14	Qian, Yisong, Liangxun Cao, Teng Guan, Lan Chen, Hongbo Xin, Yunman Li, Rui Zheng, and Deyue Yu, 2015	Primary cortical neurons of rat brain	0.01, 0.1, and 1 mM	24 hours pretreatment before exposure to 500 µM H2O2	N/A *In vitro* (cell culture)	H2O2-induced oxidative stress for 1h	NF-κB inhibition, MAPK (JNK, ERK) signaling suppression, Bcl-2/Bax ratio modulation	Genistein inhibits NF-κB by downregulating p65 and IkB phosphorylation, suppresses JNK/ERK pathways, increases Bcl-2/Bax ratio, and decreases caspase-9 and caspase-3 activities to reduce apoptosis.	Reduced oxidative stress, decreased ROS production, reduced apoptosis (caspase-9 and caspase-3), increased neuronal survival, and decreased NF-κB/JNK/ERK pathway acti	[9]
15	Banecka-Majkutewicz, Zyta, Leszek Kadziński, Michał Grabowski, Sylwia Bloch, Rajmund Kaźmierkiewicz, Joanna Jakóbkiewicz-Banecka, Magdalena Gabig-Cimińska, Grzegorz Węgrzyn, Alicja Węgrzyn, and Bogdan Banecki, 2017	*In vitro* models using enzymes and bacterial cultures (Vibrio harveyi, Bacillus subtilis)	50–100 µM Genistein	N/A	*In vitro* (bacterial cultures, enzyme assays)	Homocysteine-induced oxidative stress, inhibition of key enzymes (MetF, GPx)	Homocysteine-genistein complex formation, Methylenetetrahydrofolate reductase (MetF) inhibition, Glutathione peroxidase (GPx) modulation	Genistein directly interacts with homocysteine, forming a complex that inhibits MetF activity. Genistein alleviates homocysteine-induced GPx inhibition, reducing oxidative damage.	Reduced homocysteine-induced oxidative stress, modulation of MetF and GPx activities, formation of homocysteine-genistein complexes, potential for stroke risk reduction	[24]
16	Li, Liangdong, Jinhua Xue, Ruizhen Liu, Xiao Li, Lijuan Lai, Jiali Xie, Zhihua Huang, and Cheng Huang, 2017	*In vivo:* Male Sprague–Dawley rats (MCAO model) Adult male *In vitro*: primary rat cortical neurons (*in vitro*)	*In vivo:* 1 mg/kg and 2 mg/kg GSS *In vitro:* 0.01–1 mM GSS (key mechanistic work at 0.3 mM)	Administered 10 minutes post-MCAO surgery	Intravenous injection	*In vivo:* transient focal cerebral ischemia (2-hour occlusion followed by reperfusion) *In vitro:* 100 µM glutamate excitotoxicity	Bcl-2/Bax ratio modulation, Caspase-3 inhibition, LDH activity	GSS increases the Bcl-2/Bax ratio, indicating increased anti-apoptotic signaling. It also reduces Caspase-3 activity and LDH release, which are associated with apoptosis and cell injury, providing neuroprotection by preventing neuronal death and reducing infarct volume.	Reduced infarct size, decreased neurological deficit scores, lower Caspase-3 activity, increased Bcl-2/Bax ratio, and reduced LDH release in both *in vitro* and *in vivo* experiments.	[25]
17	Morán, Javier, Marcos Perez-Basterrechea, Pablo Garrido, Elena Díaz, Ana Alonso, Jesús Otero, Enrique Colado, and Celestino González, 2017	*In vitro* model of recurrent stroke using HT22 hippocampal neuronal cell line	1 μM for 1 h	Treatment began 1 hour after the start of first OGD, continuing through reoxygenation	N/A: *In vitro* (cell culture)	Recurrent stroke (two consecutive cycles of oxygen-glucose deprivation/reoxygenation (OGD/R))	Mitochondrial pathway, ROS production, HIF-1α regulation, Autophagy pathway (LC3)	Mitochondrial protection: prevents cytochrome c oxidase dysfunction. Apoptosis reduction: decreases PARP-1 cleavage and caspase-3 activation. HIF-1α stabilization: promotes cell survival under hypoxic conditions. Autophagy modulation: regulates autophagic activity through LC3-II/LC3-I ratio changes. Limited effect on ROS reduction.	Improved cell viability, reduced PARP-1 cleavage, decreased cytochrome c oxidase dysfunction, and enhanced mitochondrial function during recurrent ischemic injury	[26]
18	Rajput, Mithun Singh, Purnima Dey Sarkar, and Nilesh Prakash Nirmal, 2017	Streptozotocin-induced diabetic mice	2.5, 5.0, and 10 mg/kg Genistein	Administered for 2 weeks prior to ischemia-reperfusion injury	Intraperitoneal injection (i.p., o.d.)	Global cerebral ischemia (30 min occlusion of bilateral carotid arteries, 24 hours reperfusion)	DPP-4 inhibition, GLP-1 enhancement, Mitochondria-mediated apoptosis (Caspase-3 inhibition)	Genistein inhibits DPP-4 activity, increasing GLP-1 concentration, which improves mitochondrial function. Genistein also reduces neuronal apoptosis by inhibiting Caspase-3 activity and reducing oxidative stress (TBARS) in diabetic mice.	Reduced cerebral infarct size, decreased oxidative stress (TBARS), increased glutathione levels, improved cognitive and motor performance, increased cell viability (MTT assay), and reduced apoptosis (Caspase-3 activity).	[27]
19	Wang, Yu-xiang, Kun Tian, Cong-cong He, Xue-ling Ma, Feng Zhang, Hong-gang Wang, Di An, Bin Heng, Yu-gang Jiang, and Yan-qiang Liu, 2017	PC12 cells (rat adrenal medulla cells)	30 µM	1 h, 3 h, 6 h post-OGD exposure	*In vitro* (PC12 cells)	Oxygen-glucose deprivation (OGD)	Glutamate receptor subunit 2 (GluR2) modulation, potassium current restoration, AMPA receptor signaling	Genistein reduces GluR2 expression loss and restores voltage-activated potassium currents disrupted by OGD, preventing neuronal death by inhibiting apoptosis through AMPA receptor signaling modulation.	Increased cell viability, reduced apoptosis (TUNEL-positive cells), restored GluR2 expression, and enhanced K+ current amplitudes after ischemic injury	[28]
20	Miao, Zhong-Yan, Xu Xia, Lu Che, and Yan-Tao Song, 2018	Ovariectomized -Adult female Sprague Dawley- rat model of MCAO	10 mg/kg/day (Genistein)	2 weeks pretreatment, 72 hours after MCAO	Intraperitoneal injection	Transient focal cerebral ischemia	Nrf2/ARE pathway, ROS reduction, Caspase-3 inhibition	Genistein increases Nrf2 expression and its downstream target NQO1, promoting antioxidant defense and reducing ROS production. It also reduces apoptosis by decreasing cleaved-Caspase-3 levels, thus protecting neurons from ischemic injury.	Improved neurological outcomes, reduced infarct volume, decreased ROS levels, increased Nrf2 and NQO1 expression, reduced cleaved-Caspase-3 expr	[1]
21	Xue, Jinhua, Xiao Li, Jiali Xie, Ruizhen Liu, Cheng Huang, Zhihua Huang, Liangdong Li, and Lu Zhang, 2018	Adult male Sprague–Dawley rats, subjected to middle cerebral artery occlusion/reperfusion (MCAO/R)	0.5, 1.0, and 2.0 mg/kg Genistein-3’-sodium sulfonate (GSS)	Administered 10 minutes post-ischemia	Sublingual intravenous injection	Transient focal cerebral ischemia (MCAO for 2 hours followed by reperfusion)	Antioxidant defense (SOD, CAT, GSH-Px), Nitric oxide synthase (tNOS, cNOS, iNOS) regulation, Reduction of lipid peroxidation (MDA)	Genistein enhances SOD, GSH-Px, and CAT activities, reduces MDA levels, and modulates the NOS system by increasing tNOS and cNOS while reducing iNOS activity, promoting neuroprotection through antioxidant defense and nitric oxide balance.	Improved antioxidant activity, reduced MDA levels, increased neuronal viability, decreased LDH release, and modulation of the NOS system (increased tNOS and cNOS, decreased iNOS activity).	[29]
22	Lu, Li-Yan, Yan Liu, Yu-Feng Gong, and Xiu-Ying Zheng, 2019	Ovariectomized adult female Sprague-Dawley rat model of MCAO	10 mg/kg genistein	2 weeks pretreatment followed by 72 hours post-ischemia	Intraperitoneal injection	Transient focal cerebral ischemia (MCAO for 1.5 hours followed by reperfusion)	PI3K-Akt-mTOR pathway, Apoptosis reduction, Neuroprotection	Genistein activates the PI3K-Akt-mTOR pathway, increasing phosphorylation of Akt and mTOR, which enhances cell survival by inhibiting apoptosis. This reduces neuronal damage and cell apoptosis in the ischemic penumbra, promoting recovery.	Improved neurological outcomes (Garcia test), reduced infarct size (TTC staining), decreased neuronal apoptosis (TUNEL staining), increased Akt/mTOR phosphorylation (Western blot).	[3]
23	Wang, Shiquan, Jin Wang, Haidong Wei, Tingting Gu, Jiajia Wang, Zhixin Wu, and Qianzi Yang, 2020	Reproductively senescent female C57BL/6J mice	10 mg/kg Genistein	2 weeks pretreatment, 24 hours post-reperfusion	Intraperitoneal injection	Transient middle cerebral artery occlusion (MCAO)	NLRP3 inflammasome pathway, inhibition of Caspase-1, reduction of inflammatory cytokines (IL-1β, IL-18, IL-6)	Genistein inhibits the NLRP3 inflammasome in microglia, reducing the expression of Caspase-1 and pro-inflammatory cytokines (TNF-α, IL-1β, IL-18, IL-6), thus preventing neuronal apoptosis and attenuating inflammation.	Reduced infarct volume, improved neurological function, decreased neuronal apoptosis (TUNEL staining), reduced expression of NLRP3, Caspase-1, and inflammatory cytokines.	[8]
24	Liu, Chaoming, Song Liu, Lijiao Xiong, Limei Zhang, Xiao Li, Xingling Cao, Jinhua Xue, Liangdong Li, Cheng Huang, and Zhihua Huang, 2021	Male Sprague-Dawley rat model of tMCAO (transient middle cerebral artery occlusion)	1.0 mg/kg/day (Genistein-3′-sodium sulfonate, GSS)	Administered 10 minutes after ischemia, 24 hours post-reperfusion	Sublingual intravenous injection	Ischemia-reperfusion (2-hour MCAO, 24 hours reperfusion)	α7nAChR-NF-κB signaling pathway, microglial M1 polarization, Neuroinflammation	Genistein-3′-sodium sulfonate (GSS) inhibits NF-κB signaling by upregulating α7nAChR, reducing microglial M1 polarization and suppressing neuroinflammation. This leads to reduced expression of pro-inflammatory cytokines (IL-1β, TNF-α), and inhibition of NF-κB in microglial cells, preventing brain injury.	Reduced infarct volume, improved neurological function, decreased microglial M1 polarization, reduced NF-κB activation, and lower IL-1β, TNF-α, IL-6 levels.	[7]
25	Xie, Jiali, Xiao Li, Limei Zhang, Chaoming Liu, Joseph Wai-Hin Leung, Peiwen Liu, Zining Yu et al, 2021	Male Sprague-Dawley rat model of MCAO	0.5 mg/kg, 1.0 mg/kg, and 2.0 mg/kg GSS	24 hours post-reperfusion	Sublingual intravenous injection	Transient MCAO (2 hours ischemia, 24 hours reperfusion)	α7nAChR-JAK2/STAT3 signaling, Neuroinflammation, Cytokine release modulation (IL-1β, IL-6, TNF-α)	GSS inhibits neuroinflammation by upregulating α7nAChR and inhibiting JAK2/STAT3 phosphorylation. This leads to reduced levels of pro-inflammatory cytokines (IL-1β, TNF-α, IL-6), thus protecting neurons from ischemic damage.	Reduced infarct volume, improved neurological scores, reduced pro-inflammatory cytokines (IL-1β, TNF-α, IL-6), decreased JAK2/STAT3 activation, and enhanced neuronal survival.	[4]
26	Li Yuan, Zhang Jin-Jia, Chen Ru-Jia, Chen Ling Chen, Chen Su, Yang Xiao Fei and Min Jia-Wei, 2022	Neonatal mouse model of hypoxic-ischemic brain damage (HIBD)	10 mg/kg/day Genistein	3 days before hypoxia-ischemia.(HI) induction, 1 dose immediately post- HI	Intraperitoneal injection	hypoxia-ischemia (HI) brain injury model induced by combining systemic hypoxia with right common carotid artery occlusion.	Nrf2/ HO-1 anti-oxidative signaling Nrf2/HO-1 pathway for antioxidant defense and the NF-κB pathway for inflammation response	Genistein activates the Nrf2/HO-1 antioxidant pathway while inhibiting the NF-κB pathway, reducing oxidative stress and inflammation. This leads to reduced neuronal apoptosis and decreased brain infarct volume.	Reduced cerebral infarct volume, decreased oxidative stress (MDA, GSH levels), reduced inflammatory cytokines (TNF-α, IL-1β, IL-6), increased neuronal survival (TUNEL, H&E staining), improved neurological outcomes	[30]
27	Wang, Shiquan, Zhen Zhang, Jin Wang, Lina Ma, Jianshuai Zhao, Jiajia Wang, Zongping Fang, Wugang Hou, and Haiyun Guo, 2022	Ovariectomized adult female C57BL/6J mice (MCAO model of ischemic stroke)	10 mg/kg	3 doses: 6 h, 24 h, 48 h post-reperfusion	Intraperitoneal injection	Ischemia/Reperfusion (60-min MCAO followed by reperfusion)	G protein-coupled estrogen receptor (GPER)/ peroxisome proliferator-activated receptor gamma coactivator 1-alpha (PGC-1α)/ NLRP3 inflammasome (GPER/ PGC-1α/ NLRP3 inflammasome)	Genistein upregulates neuronal GPER, which in turn activates PGC-1α. This activation suppresses NLRP3 inflammasome assembly and function, leading to decreased caspase-1 activation and reduced production of proinflammatory cytokines	Reduced infarct volume, neurological deficits, neuronal apoptosis (cleaved caspase-3 and TUNEL-positive neurons), and proinflammatory cytokine levels (IL-1β, IL-18, TNF-α, IL-6) in the ischemic penumbra of ovariectomized mice following genistein treatment.	[31]
28	Xie, Ting, Liyan Shuang, Gaigai Liu, Shanshan Zhao, Zhidong Yuan, Hao Cai, Lixia Jiang, and Zhihua Huang, 2023	Neonatal male Sprague Dawley rat model of hypoxic-ischemic brain injury	0.01 mg/mL, 0.03 mg/mL and 0.1 mg/mL GSS	30 min pre-treatment and post-HI	Intraperitoneal injection	Hypoxia-Ischemia (HI) induced brain injury	NF-κB signaling, Glutamatergic synapse, Phagosome, Complement and coagulation cascades	GSS inhibits NF-κB signaling and downregulates pro-inflammatory pathways. It reduces neuronal degeneration and inflammation through modulation of complement and phagosome pathways and glutamatergic synapse regulation.	Reduced cerebral infarct volume, improved neurological outcomes, decreased neuroinflammation, reduced neuronal apoptosis and degeneration, and upregulated antioxidant defense pathways.	[32]
29	Liu, Ruizhen, Yunling Yu, Qinglian Ge, Ruixue Feng, Guixiang Zhong, Li Luo, Zun Han, Wang, Tianyun., Huang, Cheng., Xue, Jinhua. and Huang, Zhihua, 2024	Rat model of transient MCAO	1 mg/kg/day (Genistein-3′-sodium sulfonate, GSS)	28 days post-MCAO	Intraperitoneal injection	Transient focal cerebral ischemia	NF-κB signaling, Astrocyte polarization, Neuroinflammation	GSS inhibits the NF-κB signaling pathway, reduces the pro-inflammatory A1 astrocyte phenotype while promoting the anti-inflammatory A2 astrocyte phenotype, thus reducing neuroinflammation and enhancing brain functional recovery.	Improved cognitive and motor functions, reduced pro-inflammatory cytokines (IL-1β, TNF-α), increased neurotrophic factors (BDNF, IL-10), suppressed astrocyte activation and polarization to A1, promoted A2 polarization.	[33]
30	Li, Li, Saisai Liu, Mengzhe Wang, Mengjia Li, Yi Liu, Haili Chen, Jie Chen, Weiting Tao, Li Huang, and Shidi Zhao, 2025	*In vivo*: Male Sprague-Dawley rats (MCAO model) *In vitro:* PC12 cells (OGD/R model)	*In vivo:* 25, 50, 100 mg/kg *In vitro:* 10, 20, 30 µM	*In vivo:* 21 days pretreatment *In vitro*: 24 h pretreatment	p.o. (intragastric administration)	*In vivo:* cerebral ischemia/reperfusion injury (MCAO model in rats) *In vitro:* OGD/reoxygenation (OGD/R in PC12 cells	Wnt/Ca² ⁺ signaling pathway (Wnt5a, Frizzled-2, p-CaMKII, IP₃R)	Genistein inhibits the Wnt/Ca² ⁺ signaling pathway by downregulating Wnt5a and Frizzled-2, reducing intracellular calcium release via IP₃R, suppressing oxidative stress (↓ROS, ↓ NOX1), and enhancing antioxidant response (↑SOD1, ↑ SOD2), ultimately preventing apoptosis.	Reduced infarct volume and brain edema; improved neurological scores; decreased calcium overload, ROS, and apoptosis in ischemic penumbra and PC12 cells; validated mechanistic role via Frizzled-2 knockdown	[34]
31	Yu, Yunling, Xinglan Liao, Kehui Xing, Ziyu Xie, Ningyuan Xie, Yinwen He, Zhihua Huang, Xiaolu Tang, and Ruizhen Liu, 2025	Adult male Sprague-Dawley rats subjected to tMCAO	2 mg/kg GSS	Daily for 7 days post-reperfusion	Intraperitoneal injection	Transient cerebral ischemia	GPER1/ NLRP3 inflammasome	GSS activates GPER1, which suppresses NLRP3 inflammasome activation. This leads to reduced levels of pyroptosis-related markers including ASC, cleaved-caspase-1, GSDMD-N, IL-1β, and IL-18. The inhibition of NLRP3 prevents cell membrane perforation and pro-inflammatory cytokine release, thereby attenuating pyroptosis and neuroinflammation.	Reduced expression of NLRP3, ASC, cleaved-caspase-1, GSDMD-N, IL-1β, and IL-18; decreased NLRP3 + /NeuN+ cells in ischemic penumbra; improved motor function and reduced anxiety-like behavior (OFT); neuroinflammation suppressed; therapeutic effects reversed upon GPER1 inhibitor (G15) administration	[35]

**Abbreviations:** ALS: Amyotrophic Lateral Sclerosis; ARE: Antioxidant Response Element; BDNF: Brain-Derived Neurotrophic Factor; CAT: Catalase; CXCL1: C-X-C Motif Chemokine Ligand 1; DPP-4: Dipeptidyl Peptidase-4; ERCC2: Excision Repair Cross-Complementation Group 2; ERK: Extracellular Signal-Regulated Kinase; ERK1/2: ERK isoforms 1 and 2; GLP-1: Glucagon-Like Peptide-1; GPER: G Protein-Coupled Estrogen Receptor (GPER); GR: Glucocorticoid Receptor; GSH: Glutathione; HIF-1α: Hypoxia-Inducible Factor 1-alpha; HO-1: Heme Oxygenase 1; IL-13: Interleukin 13; IL-1β: Interleukin 1 Beta; IL-2: Interleukin 2; IL-6: Interleukin 6; JAK2: Janus Kinase 2; JNK: c-Jun N-terminal Kinase; LC3: Microtubule-associated proteins 1A/1B light chain 3; LDH: Lactate Dehydrogenase; MAPK: Mitogen-Activated Protein Kinase; MCA: Middle Cerebral Artery; MDA: Malondialdehyde; Mdm2: Mouse Double Minute 2 homolog; MetF: Methylenetetrahydrofolate Reductase; NMDA: N-Methyl-D-Aspartate; NOX: NADPH Oxidase; NQO1: NAD(P)H Quinone Dehydrogenase 1; NRF1: Nuclear Respiratory Factor 1; Nrf2: Nuclear factor erythroid 2–related factor 2; OGD: Oxygen-Glucose Deprivation; OVX: Ovariectomized; PARP-1: Poly (ADP-ribose) polymerase 1; PGC-1α: Peroxisome Proliferator-Activated Receptor Gamma Coactivator 1-Alpha; PI3K: Phosphoinositide 3-Kinase; p.o.: per os; ROS: Reactive Oxygen Species; SHR: Spontaneously Hypertensive Rats; SOD: Superoxide Dismutase; SOD1: Superoxide Dismutase 1; STAT3: Signal Transducer and Activator of Transcription 3; TNF-α: Tumor Necrosis Factor Alpha; TUNEL: Terminal deoxynucleotidyl transferase dUTP Nick-End Labeling; WKY: Wistar Kyoto Rats; cNOS: Constitutive Nitric Oxide Synthase; iNOS: Inducible Nitric Oxide Synthase; mTOR: Mammalian Target of Rapamycin; tMCAO: Transient Middle Cerebral Artery Occlusion; tNOS: Total Nitric Oxide Synthase; α7nAChR: Alpha-7 Nicotinic Acetylcholine Receptor.

### 3.1. Evidence of genistein’s mechanism in in vivo models

*In vivo* studies were performed on rat and mouse models, including models of middle cerebral artery occlusion (MCAO), global cerebral ischemia (GCI) and hypoxic-ischemic brain injury. A total of 26 studies from the final included studies contributed to the *in vivo* evidence in evaluating genistein’s protective mechanisms in ischemic stroke. These are detailed in Table 3. These studies explored the neuroprotective mechanisms of genistein or its more soluble counterpart, GSS, and consistently demonstrated reduced infarct size, improved neurological scores, and modulation of inflammation, oxidative stress, and apoptosis. Some researchers used GSS, a sulfonated version of pure genistein, to improve water solubility and bioavailability.

**Table 3:** Summary of key molecular pathways and mechanistic categories of genistein in *in vivo* models of ischemic stroke.

Study No	Pathway	Mechanism Category
1	Estrogen receptor, ROS	Anti-oxidative
2	JAK2/STAT3	Anti-inflammatory
3	Mitochondrial, NMDA receptor, ROS	Anti-apoptotic, Anti-oxidative
5	NOX, NQO1/ERCC2	Anti-oxidative
6	Estrogen receptors	Vascular-related
7	Mitochondrial, ROS- NF-κB	Anti-apoptotic, Anti-oxidative
8	Thromboxane A2	Vascular-related
9	eNOS, Nrf2/HO-1	Anti-oxidative
10	NF-κB, Cytokines	Anti-inflammatory
11	GR signaling	Anti-inflammatory
12	ERK1/2, Bcl-2/Bax	Anti-apoptotic
13	NRF1, Oxidative stress	Anti-apoptotic, Anti-oxidative
16	Bcl-2/Bax, Caspase-3, LDH	Anti-apoptotic
18	DPP-4, Mitochondrial	Anti-oxidative
20	Nrf2/ARE, Caspase-3	Anti-oxidative, Anti-apoptotic
21	Antioxidant enzymes, NOS system	Anti-oxidative
22	PI3K-Akt-mTOR	Anti-apoptotic
23	NLRP3, Caspase-1, Cytokines	Anti-inflammatory
24	α7nAChR - NF-κB	Anti-inflammatory
25	α7nAChR -JAK2/STAT3	Anti-inflammatory
26	Nrf2/HO-1, NF-κB	Anti-inflammatory, Anti-oxidative
27	GPER, PGC-1α, NLRP3 inflammasome	Anti-inflammatory, Anti-pyroptotic
28	NF-κB, Synaptic, Phagosome	Anti-inflammatory, Anti-oxidative
29	NF-κB, Astrocytes	Anti-inflammatory
30	Wnt5a, Frizzled-2, IP₃R, SOD1/SOD2	Anti-oxidative, Anti-apoptotic
31	GPER1, NLRP3 inflammasome, GSDMD-N	Anti-inflammatory, Anti-pyroptotic

### 3.2. Evidence of genistein’s mechanism in in vitro models

Seven studies examined genistein’s neuroprotective mechanisms using *in vitro* models, including various neuron-like and cortical cell lines. These are detailed in Table 4. These studies demonstrated genistein’s ability to reduce oxidative stress, regulate apoptotic proteins, and improve cell viability. GSS, though primarily applied *in vivo*, also showed efficacy in cell-based assays when used.

**Table 4:** Summary of key molecular pathways and mechanistic categories of genistein in *in vitro* models of ischemic stroke.

Study No	Pathway	Mechanism Category
4	ERs, PI3K, MAPK/ERK	Anti-apoptotic
14	NF-κB, JNK, ERK, Bcl-2/Bax	Anti-apoptotic, Anti-oxidative
15	MetF, GPx	Anti-oxidative
16	Bcl-2/Bax, Caspase-3, LDH	Anti-apoptotic
17	Mitochondrial, HIF-1Î ± , Autophagy	Anti-apoptotic, Anti-oxidative
19	GluR2, AMPA signaling	Anti-apoptotic
30	Wnt5a, Frizzled-2, IP₃R, SOD1/SOD2	Anti-oxidative, Anti-apoptotic

### 3.3. Critical appraisal/ Quality

The quality scores of the included studies ranged from 8 to 10, based on the modified McMaster Review Form. The average quality score was 8.39. Detailed scoring per study is shown in Table 5.

**Table 5:** Modified McMaster critical review form for quantitative studies for individual studies.

Author, Year	Level of evidence	Total score	1	2	3	4	5	6	7	8	9	10	11
Trieu, Vuong N.and Fatih M. Uckun 1999	3	8	1	1	1	0	1	1	1	1	0	0	1
Li, Hong-Chun and Zhang, Guang-Yi 2003	3	8	1	1	1	0	1	1	1	1	0	0	1
Liang, Hua-Wei, Shui-Feng Qiu, Jia Shen, Li-Na Sun, Jing-Ye Wang, Iain C. Bruce, and Qiang Xia. 2008	3	9	1	1	1	0	1	1	1	1	0	1	1
Schreihofer, Derek A. and Lori Redmond, 2009	3	9	1	1	1	0	1	1	1	1	1	0	1
Ma, Yulin, Jennifer C. Sullivan, and Derek A. Schreihofer, 2010	3	8	1	1	1	0	1	1	1	1	0	0	1
Castello-Ruiz M, Torregrosa G, Burguete M, Salom J, Gil J, Miranda F… et al. 2011	3	8	1	1	1	0	1	1	1	1	0	0	1
Qian, Yisong, Liangxun Cao, Teng Guan, Lan Chen, Hongbo Xin, Yunman Li, Rui Zheng, and Deyue Yu, 2012	3	8	1	1	1	0	1	1	1	1	0	0	1
Cortina, Belén, Germán Torregrosa, María Castelló-Ruiz, María C. Burguete, Antonio Moscardó, Ana Latorre, Juan B. Salom, Juana Vallés, María T. Santos, and Enrique Alborch, 2013	3	9	1	1	1	0	1	1	1	1	1	0	1
Wang, Ruimin, Jingyi Tu, Quanguang Zhang, Xi Zhang, Ying Zhu, Wendong Ma, Cheng Cheng, Darrell W. Brann, and Fang Yang, 2013	3	9	1	1	1	0	1	1	1	1	0	1	1
Shambayati, Maryam, Maharshi Patel, Yulin Ma, Rebecca L. Cunningham, and Derek A. Schreihofer, 2014	3	8	1	1	1	0	1	1	1	1	0	0	1
Shi, Rengfei, Shunli Wang, Xiang Qi, Si Chen, Peijie Chen, and Quanguang Zhang, 2014	3	8	1	1	1	0	1	1	1	1	0	0	1
Wang, Shiquan, Haidong Wei, Min Cai, Yan Lu, Wugang Hou, Qianzi Yang, Hailong Dong, and Lize Xiong, 2014	3	9	1	1	1	0	1	1	1	1	0	1	1
Aras, Adem Bozkurt, Mustafa Guven, Tarik Akman, Hasan Alacam, Yildiray Kalkan, Coskun Silan, and Murat Cosar, 2015	3	8	1	1	1	0	1	1	1	1	0	0	1
Qian, Yisong, Liangxun Cao, Teng Guan, Lan Chen, Hongbo Xin, Yunman Li, Rui Zheng, and Deyue Yu, 2015	3	8	1	1	1	0	1	1	1	1	0	0	1
Banecka-Majkutewicz, Zyta, Leszek Kadziński, Michał Grabowski, Sylwia Bloch, Rajmund Kaźmierkiewicz, Joanna Jakóbkiewicz-Banecka, Magdalena Gabig-Cimińska, Grzegorz Węgrzyn, Alicja Węgrzyn, and Bogdan Banecki, 2017	3	7	1	1	0	0	1	1	1	1	0	0	1
Li, Liangdong, Jinhua Xue, Ruizhen Liu, Xiao Li, Lijuan Lai, Jiali Xie, Zhihua Huang, and Cheng Huang, 2017	3	8	1	1	1	0	1	1	1	1	0	0	1
Morán, Javier, Marcos Perez-Basterrechea, Pablo Garrido, Elena Díaz, Ana Alonso, Jesús Otero, Enrique Colado, and Celestino González, 2017	3	7	1	1	1	0	1	1	0	1	0	0	1
Rajput, Mithun Singh, Purnima Dey Sarkar, and Nilesh Prakash Nirmal, 2017	3	9	1	1	1	0	1	1	1	1	1	0	1
Wang, Yu-xiang, Kun Tian, Cong-cong He, Xue-ling Ma, Feng Zhang, Hong-gang Wang, Di An, Bin Heng, Yu-gang Jiang, and Yan-qiang Liu, 2017	3	8	1	1	1	0	1	1	1	1	0	0	1
Miao, Zhong-Yan, Xu Xia, Lu Che, and Yan-Tao Song, 2018	3	10	1	1	1	1	1	1	1	1	1	0	1
Xue, Jinhua, Xiao Li, Jiali Xie, Ruizhen Liu, Cheng Huang, Zhihua Huang, Liangdong Li, and Lu Zhang, 2018	3	8	1	1	1	0	1	1	1	1	0	0	1
Lu, Li-yan, Yan Liu, Yu-feng Gong, and Xiu-ying Zheng, 2019	3	10	1	1	1	1	1	1	1	1	1	0	1
Wang, Shiquan, Jin Wang, Haidong Wei, Tingting Gu, Jiajia Wang, Zhixin Wu, and Qianzi Yang, 2020	3	8	1	1	1	0	1	1	1	1	0	0	1
Liu, Chaoming, Song Liu, Lijiao Xiong, Limei Zhang, Xiao Li, Xingling Cao, Jinhua Xue, Liangdong Li, Cheng Huang, and Zhihua Huang, 2021	3	8	1	1	1	0	1	1	1	1	0	0	1
Xie, Jiali, Xiao Li, Limei Zhang, Chaoming Liu, Joseph Wai-Hin Leung, Peiwen Liu, Zining Yu et al, 2021	3	8	1	1	1	0	1	1	1	1	0	0	1
Li Yuan, Zhang Jin-Jia, Chen Ru-Jia, Chen Ling Chen, Chen Su, Yang Xiao Fei and Min Jia-Wei, 2022	3	9	1	1	1	1	1	1	1	1	0	0	1
Wang, Shiquan, Zhen Zhang, Jin Wang, Lina Ma, Jianshuai Zhao, Jiajia Wang, Zongping Fang, Wugang Hou, and Haiyun Guo, 2022	3	10	1	1	1	0	1	1	1	1	1	1	1
Xie, Ting, Liyan Shuang, Gaigai Liu, Shanshan Zhao, Zhidong Yuan, Hao Cai, Lixia Jiang, and Zhihua Huang, 2023	3	8	1	1	1	0	1	1	1	1	0	0	1
Liu, Ruizhen, Yunling Yu, Qinglian Ge, Ruixue Feng, Guixiang Zhong, Li Luo, Zun Han, Wang, Tianyun., Huang, Cheng., Xue, Jinhua. and Huang, Zhihua 2024	3	10	1	1	1	1	1	1	1	1	1	0	1
Li, Li, Saisai Liu, Mengzhe Wang, Mengjia Li, Yi Liu, Haili Chen, Jie Chen, Weiting Tao, Li Huang, and Shidi Zhao, 2025	3	8	1	1	1	0	1	1	1	1	0	0	1
Yu, Yunling, Xinglan Liao, Kehui Xing, Ziyu Xie, Ningyuan Xie, Yinwen He, Zhihua Huang, Xiaolu Tang, and Ruizhen Liu, 2025	3	10	1	1	1	0	1	1	1	1	1	1	1

**Note:** Level of evidence: 3 = non-experimental, correlational, or cohort study. Critical appraisal scoring criteria: 1 = study purpose clearly stated; 2 = relevant literature reviewed; 3 = sample thoroughly described; 4 = sample size justified; 5 = reliable outcome measures; 6 = valid outcome measures; 7 = results presented with statistical significance; 8 = appropriate analysis methods; 9 = educational relevance discussed; 10 = dropouts reported; 11 = conclusions deemed appropriate.

### 3.4. Inter-rater reliability assessment

To measure the inter-rater reliability between these reviewers for the critical appraisal of the final included studies, Cohen’s Kappa statistic was used. Cohen’s Kappa agreement values were calculated to assess the level of agreement between the two reviewers (N.Y.M.K and N.A.). If there were any disagreements, then it was solved by third and fourth reviewers (R.S. and A.M.A.). Cohen’s Kappa value (κ) for the critical appraisal of a total of 31 final eligible articles was reviewed, and the value obtained was κ = 0.829, which was almost perfect agreement. The inter-rater reliability tables were given as S1 Table.

## 4. Discussion

This systematic review identified several key mechanisms through which genistein exerts neuroprotective effects in ischemic stroke models. Across both *in vivo* and *in vitro* studies, genistein consistently demonstrated pleiotropic actions encompassing three major categories: anti-apoptotic, anti-inflammatory, and antioxidative mechanisms. Many of the included studies investigated overlapping pathways, reflecting genistein’s capacity to target multiple aspects of neuronal injury. These mechanisms were demonstrated in various experimental conditions, including *in vitro* models involving neuronal exposure to oxidative stressors such as OGD, and glutamate toxicity, as well as *in vivo* models of cerebral ischemia such as MCAO and GCI. The following sections synthesize the mechanistic evidence derived from these models, beginning with genistein’s anti-apoptotic effects, followed by its anti-inflammatory and antioxidative roles in ischemic stroke.

### 4.1. Anti-apoptotic mechanism of genistein in ischemic stroke

Genistein has been shown to exhibit anti-apoptotic effects on *in vivo* and *in vitro* models of ischemic stroke. The mechanisms include regulation of the mitochondria-mediated apoptotic pathway, regulation of the Bcl-2/Bax ratio and caspase activation inhibition. The use of *in vivo* animal models has demonstrated that genistein is effective in inhibiting apoptosis via mitochondrial and receptor-mediated pathways. Liang et al. [15] demonstrated in a rat model of transient global cerebral ischemia that genistein suppressed mitochondrial ROS and lipid peroxidation, inhibited cytosolic cytochrome c and caspase-3 activity, and reduced TUNEL-positive neurons, indicating that the mitochondria-dependent apoptotic pathway had been suppressed. Mitochondrial integrity and Bcl-2 family proteins are known to play a significant role in regulating caspase-3-mediated neuronal apoptosis during ischemia [36]. Equally, Qian et al. [6] found that genistein dramatically reduced infarct size and oxidative stress markers by inhibiting mitochondrial ROS generation and NF-κB activation, thereby reducing neuronal apoptosis.

Genistein’s anti-apoptotic effect is further enhanced by hormonal and kinase modulation. Wang et al. [22] also reported that in ovarizing mice, genistein stimulated ERK1/2 phosphorylation, increased Bcl-2, decreased Bax, and enhanced neurological performance; which were inhibited by ERK. Similarly, Aras et al. [23] demonstrated that genistein minimized oxidative stress and apoptosis in MCAO rats by increasing the expression of NRF1 and SOD, and decreasing the activity of caspase-3 and −9.

In line with those results, Miao et al. [1] identified that genistein stimulated the Nrf2/NQO1 antioxidant pathway, reduced ROS, and increased neuronal survival, which is in line with the contribution of the Nrf2/HO-1 pathway to alleviating ischemic apoptosis [37]. Lu et al. [3] further added that genistein stimulated PI3K-Akt-mTOR signaling, which increased the phosphorylation of Akt and mTOR and reduced infarct size and neuronal death. The neuroprotective role of PI3K/Akt signaling in stroke has been corroborated in other ischemia models [38]. Li et al. [34] showed that genistein inhibited Wnt/Ca^2+^, reducing ROS and NOX1 and increasing antioxidant enzyme.

These observations are reinforced by complementary *in vitro* studies, which explain the upstream molecular events. Schreihofer and Redmond [16] observed that in cortical neurons subjected to OGD toxicity or glutamate toxicity, estrogen receptor-dependent PI3K and ERK signaling by genistein inhibited LDH release and caspase activity. Qian et al. [9] validated that genistein prevented H₂O₂-induced neuronal apoptosis through the restoration of Bcl-2/Bax ratio, caspase-9 and −3 inhibition, and the inhibition of NF-κB, JNK and ERK phosphorylation. In models of *in vitro* studies, genistein steadily decreased neuronal apoptosis by stabilizing mitochondrial and calcium homeostasis. It has increased the anti-apoptotic ratio of Bcl-2/Bax and inhibited caspase-3 activity [25], reduced ROS generation and cleaving of the proteins of the proteolytic enzyme PARP-1 and increased autophagic balance by modulating LC3, avoiding calcium overload and recovered the expression and potassium currents of the AMPA receptor, GluR2 [28]. Collectively, these multi-model results demonstrate the convergent anti-apoptotic mechanism of genistein, including mitochondrial stabilization, control of pro-survival kinase signatures (ERK1/2, PI3K/Akt/mTOR), Ca^2+^ and oxidative homeostasis, and caspase activation. The mechanistic consistency in the *in vitro* and *in vivo* whole systems testifies to the strong neuroprotective potential of genistein in ischemic stroke.

Beyond the studies included in this review, additional literature supports genistein’s anti-apoptotic role in other organ systems. In a rat model of polycystic ovarian syndrome, genistein enhanced Bcl-2 expression and suppressed Bax in ovarian granulosa cells [39]. In the cardiovascular system, Hu et al. [40] showed that genistein protected H9c2 cardiomyoblasts from isoproterenol-induced mitochondrial apoptosis by down-regulating pro-apoptotic proteins (Bad, caspase-3, −8, −9) and up-regulating survival pathways including Akt, ERK1/2, and NF-κB. Similarly, Si and Liu [41] reported that genistein reduced TNF-α-induced apoptosis in human aortic endothelial cells by activating the p38β MAPK pathway and upregulating Bcl-2, highlighting its vasculoprotective role. These findings across endocrine, cardiovascular, and vascular models reinforce genistein’s capacity to inhibit apoptosis through multiple signaling mechanisms, supporting its broader therapeutic relevance in diseases characterized by oxidative or stress-induced cellular damage.

### 4.2. Anti-inflammatory mechanism of genistein in ischemic stroke

Genistein has demonstrated significant anti-inflammatory properties in various *in vivo* models of ischemic stroke, primarily through its modulation of multiple key signaling pathways. Early evidence from Li and Zhang [14] revealed that genistein inhibited STAT3 phosphorylation and DNA-binding activity in the rat hippocampus following cerebral ischemia/reperfusion, indicating direct blockade of a major inflammatory transcription factor. A soy isoflavone-enriched diet was shown to inhibit NF-κB translocation in ovariectomized rats subjected to tMCAO, leading to the suppression of pro-inflammatory cytokines, including IL-1β, IL-2, IL-13, and CXCL1, and a reduction in microglial activation within the infarct core [20]. In another study using a forebrain ischemia model, genistein attenuated neuroinflammation by inhibiting GR nuclear translocation and promoting its degradation via the Mdm2-ubiquitin pathway, thus preventing GR-induced microglial activation and neuronal injury in the hippocampus [21].

In reproductively senescent mice, genistein reduced neuroinflammation by inhibiting the NLRP3 inflammasome, resulting in decreased expression of Caspase-1 and inflammatory cytokines, such as IL-1β, IL-6, and IL-18, alongside improved neurological outcomes [8]. GSS, a water-soluble derivative, further enhanced anti-inflammatory efficacy by upregulating the α7nAChR, leading to the inhibition of NF-κB and a shift away from pro-inflammatory M1 microglial polarization. This was associated with reduced infarct volume and improved neurological function [7].

Likewise, Wang et al. [31] also demonstrated that genistein increased neuronal GPER, which stimulated PGC-1α and thus inhibited NLRP3 inflammasome assembly in ovariectomized ischemic mice. This cascade decreased caspase-1 and pro-inflammatory cytokine release and yielded smaller infarcts, better neurological outcome and reduced apoptotic neurons- identifying a GPER/PGC-1alpha-dependent anti-inflammatory mechanism between hormonal and mitochondrial regulation in ischemic neuroprotection.

Moreover, GSS also attenuated neuroinflammation via suppression of JAK2/STAT3 signaling and cytokine release, including IL-1β, IL-6, and TNF-α, in MCAO models [4]. In HIBD, genistein activated the Nrf2/HO-1 antioxidative pathway while inhibiting NF-κB, reducing both oxidative stress and inflammation, and promoting neuronal survival [30]. Similar effects were observed with GSS, which downregulated multiple pro-inflammatory cascades including complement, phagosome, and glutamatergic synapse pathways, resulting in decreased neuronal degeneration and neuroinflammation [32]. Notably, long-term treatment with GSS in post-MCAO rats not only suppressed NF-κB but also promoted a beneficial shift in astrocyte polarization from the pro-inflammatory A1 phenotype to the neuroprotective A2 phenotype, along with increased levels of IL-10 and BDNF and improved cognitive and motor recovery [33]. Most recently, Yu et al. [35] demonstrated that GSS prevented NLRP3-mediated pyroptosis following ischemia by reducing the expression of cleaved-caspase-1, GSDMD-N, IL-1β, and IL-18, thereby limiting inflammatory cell death and restoring neurological function.

Collectively, these findings suggest that genistein and its derivatives exert robust *in vivo* anti-inflammatory effects through inhibition of NF-κB, STAT3, and GR signaling, suppression of NLRP3 inflammasome activation and pyroptosis, and modulation of glial polarization. By simultaneously targeting transcriptional, inflammasome, and glial-cell pathways, genistein offers a multi-targeted strategy for mitigating post-ischemic neuroinflammation.

Beyond the studies included in our systematic review, external mechanistic literature further supports the plausibility of genistein’s anti-inflammatory actions through multiple signaling pathways. These findings are consistent with other *in vivo* studies showing that genistein suppresses NF-κB signaling and pro-inflammatory cytokine release in models of systemic or organ-specific inflammation, such as high-fat diet-induced liver inflammation [42] and LPS-induced acute lung injury [43], further supporting its potential to mitigate neuroinflammation through similar mechanisms.

Moreover, genistein’s anti-inflammatory effects have been linked to inhibition of the NLRP3 inflammasome and modulation of downstream pathways. In a mouse model of dextran sulfate sodium (DSS)-induced colitis, genistein significantly suppressed NLRP3 inflammasome activation via the TGR5–cAMP signaling axis, leading to reduced IL-1β secretion and improved clinical outcomes [44]. Similarly, in a traumatic brain injury (TBI) model, genistein alleviated neurological deficits and anxiety-like behaviors by downregulating NLRP3 and caspase-1 expression, suggesting a central role in neuroinflammatory regulation [45].

Additionally, genistein has been shown to activate peroxisome proliferator-activated receptor gamma (PPARγ), a nuclear receptor involved in regulating both metabolic and inflammatory genes. For example, in human mesenchymal stem cells, genistein upregulated PPARγ expression, confirming its role as a functional activator of this pathway [46]. Although demonstrated in the context of adipogenesis, this mechanism aligns with PPARγ’s known anti-inflammatory effects in other cell types.

Complementing this, genistein has also demonstrated vascular anti-inflammatory activity through cAMP preservation and inhibition of monocyte adhesion under hyperglycemic conditions. While not directly mediated via PPARγ, these actions are consistent with anti-inflammatory profiles typically associated with PPARγ activation [47].

### 4.3. Anti-oxidative mechanism of genistein in ischemic stroke

Genistein exhibits strong antioxidative activity in both *in vivo* and *in vitro* models of cerebral ischemia, oxidative stress, and hypoxic brain injury. In one of the earliest studies, Trieu and Uckun demonstrated that genistein reduced hydroxyl radical formation and oxidative damage in murine models of familial ALS and photochemically induced stroke, primarily via tyrosine kinase inhibition and estrogen receptor-dependent pathways [13]. Similarly, Liang et al. showed that in rats subjected to transient global cerebral ischemia, genistein significantly attenuated mitochondrial ROS production, reduced lipid peroxidation as evidenced by decreased MDA levels, and suppressed caspase-3 activation, highlighting its role in preserving mitochondrial function under oxidative conditions [15].

Ma et al. further confirmed genistein’s ability to mitigate oxidative stress through estrogen receptor-mediated mechanisms in male and ovariectomized female rats undergoing transient tMCAO [17]. Genistein reduced NADPH oxidase activity, lowered superoxide levels, and upregulated NQO1 and ERCC2, genes critical for antioxidant defense and DNA repair [17]. In mice treated orally with genistein for two weeks, Qian et al. reported a dose-dependent reduction in oxidative stress, as evidenced by decreased levels of MDA and ROS, along with increased activity of endogenous antioxidant enzymes such as SOD and GPx [6].

A mechanistically distinct yet complementary pathway was described by Wang et al., who demonstrated that genistein activated the eNOS/Nrf2/HO-1 axis, promoting the nuclear translocation of Nrf2 and enhancing the expression of HO-1, which collectively contributed to reduced oxidative injury in a rat model of global cerebral ischemia [19]. In another study, Aras et al. demonstrated that genistein administration in MCAO-induced rats led to increased NRF1 expression and SOD activity, reduced MDA levels, and protected against neuronal degeneration by preserving mitochondrial function [23]. In a diabetic stroke model, Rajput et al. noted genistein’s role in inhibiting DPP-4 activity and boosting GLP-1 levels, which subsequently improved mitochondrial integrity, reduced oxidative stress (TBARS), and increased glutathione concentrations [27].

Miao et al. and Li et al. both reported that genistein significantly upregulated the Nrf2/ARE antioxidant signaling pathway, enhanced expression of NQO1, and reduced ROS levels, contributing to improved neurological recovery after ischemic insult [1,30]. Additionally, Xue et al. showed that GSS not only enhanced SOD, CAT, and GPx activity but also regulated the nitric oxide synthase (NOS) system, increasing total and constitutive NOS while suppressing iNOS, thus optimizing redox balance in ischemic brain tissue [29].

*In vitro* findings further corroborate these results. Qian et al. demonstrated that genistein pretreatment of primary cortical neurons exposed to H₂O₂ reduced ROS levels, suppressed NF-κB and MAPK (JNK/ERK) activation, and increased the Bcl-2/Bax ratio, indicating enhanced cellular resilience to oxidative insult [9]. Banecka-Majkutewicz et al. provided additional mechanistic insights by showing that genistein formed complexes with homocysteine and restored GPx activity, mitigating homocysteine-induced oxidative stress in enzyme-based and bacterial models [24]. In hippocampal HT22 cells undergoing repeated oxygen-glucose deprivation/reoxygenation cycles, Morán et al. reported that genistein preserved cytochrome c oxidase activity, stabilized HIF-1α, reduced PARP-1 cleavage, and maintained mitochondrial function, underscoring its role in protecting against oxidative damage during recurrent ischemia [26].

Collectively, these studies underscore genistein’s multifaceted antioxidative mechanisms. Its ability to inhibit ROS, activate endogenous antioxidant defenses (particularly the Nrf2/HO-1 and Nrf2/ARE pathways), protect mitochondrial integrity, and modulate nitric oxide and redox-sensitive enzymes underlines its therapeutic potential in oxidative stress-related neural injury.

Beyond the studies included in our systematic review, external mechanistic literature further supports the plausibility of genistein’s antioxidative actions in non-neural tissues through diverse pathways. For instance, in a murine model of alcoholic liver disease, genistein administration ameliorated acetaldehyde-induced oxidative stress and liver injury by restoring Nrf2 and HO-1 signaling pathway [48]. Similarly, in mice with diet-induced non-alcoholic fatty liver disease (NAFLD), genistein supplementation reduced hepatic steatosis and oxidative stress, effects attributed to improvements in visceral adipocyte metabolism and antioxidant defenses [49]. In diabetic cardiomyopathy, Gupta et al. [50] demonstrated that genistein significantly reduced oxidative stress, lipid peroxidation, and inflammatory cell infiltration in streptozotocin-induced diabetic rats, highlighting its cardioprotective role through attenuation of oxidative and inflammatory damage. Furthermore, genistein has also been shown to protect against acetaminophen-induced hepatotoxicity in mice by enhancing hepatic sirtuin 1 (SIRT1) expression and activity, thereby augmenting antioxidative defenses [51]. Collectively, these findings from non-neural tissues reinforce genistein’s multifaceted antioxidative mechanisms, highlighting its systemic therapeutic potential beyond the brain.

### 4.4. Integrated mechanistic interplay in genistein’s neuroprotective role

Several pathways of genistein have been observed throughout the considered literature as neuroprotectants in ischemic stroke. Importantly, it was demonstrated that genistein exhibits a significant overlap in its therapeutic effects on ischemic stroke models, affecting the Nrf2, NF-κB, and NLRP3 pathways. Although there is a difference in the types of models, the three pathways are biologically linked, creating a unified mechanistic structure through which genistein promotes its neuroprotective effects. These pathways, though historically classified as antioxidative or inflammatory responses, are functionally connected in a cascade.

Genistein continues to modulate the redox inflammatory balance by activating Nrf2 and inhibiting NF-κB, subsequently suppressing the NLRP3 inflammasome. Nrf2 interaction *in vivo* has been observed in global/focal/HIBD models, Nrf2/ HO-1 through eNOS activation with less oxidative damage [19], direct Nrf2- NQO1 upregulation and less ROS/apoptosis in OVX-MCAO rats [1], and simultaneous Nrf2-up/NF-κB-down signaling with less neuroinflammation in neonatal HIBD [30].

NF-κB signaling suppression proved to be one of the key mechanisms in both animal and cellular models. Genistein interfered with mitochondrial ROS-NF-κB feedback in MCAO mice [6], prevented nuclear translocation of NF-κB and cytokine release after dietary soy intake [20] and stimulated α7nAChR to inhibit NF-κB-mediated M1 microglial polarization [7]. NF-κB activity was also attenuated in neonatal hypoxic-ischemic brain injury [32] and in long-term GSS treatment, which transformed astrocytes of the pro-inflammatory A1 phenotype to the neuroprotective A2 state [33]. Additional *in vitro* evidence also showed that genistein inhibits NF-κB in combination with the JNK and ERK pathways and reinstates the Bcl-2/Bax ratio during the oxidative stressed conditions [9].

The activity of NLRP3 inflammasomes was consistently inhibited at the downstream level in various ischemic models. Genistein blocked microglial NLRP3 activation and downstream effects, caspase-1 and IL-1b, in mice undergoing reproductive senescence [8]; activated neuronal GPER1 signal to inhibit NLRP3 assembly and cytokine maturation, an effect reversed by GPER inhibition [31]; and inhibited pyroptotic cell death by suppressing GPER1-NLRP3 axis after tMCAO [35].

Together, these results outline a mechanistic cascade of events where genistein triggers Nrf2-mediated antioxidant defense mechanism, inhibits NF-κB -mediated inflammation, and suppresses NLRP3-mediated pyroptosis. This tri-pathway cascade, characterized by two-way crosstalk between the oxidative stress response and inflammatory response, points to an integrated signaling axis where enhanced Nrf2 activity attenuates oxidative stress and thereby suppresses NF-κB activation, which in turn prevents NLRP3 inflammasome assembly, underlying the coordinated neuroprotective effects of genistein in models of ischemic stroke [9,10,14,20,23,24,26,29,31].

Reoccurrence of other signaling pathways, including PI3K/Akt, ERK1/2, Bcl-2/Bax and JAK/STAT, can be explained by two major factors. Firstly, genistein has a dual effect, acting as both an estrogen receptor-based signaling molecule and a tyrosine kinase blocker, which enables it to influence several survival-related pathways. Second, although studies are methodologically diverse, including a variety of ischemia models, e.g., MCAO, GCI, or OGD, and different dosages or routes of administration are used, the pathological hallmarks of ischemic injury, such as oxidative stress, inflammation, and apoptosis, are consistent.

These common pathophysiological events naturally intersect on conserved molecular signaling hubs, which are recurrently studied in various studies. Instead of suggesting redundancy, the recurrent finding of these signaling pathways indicates the conserved biopharmacodynamics of genistein and biologically denotes its importance in the ischemic stroke model.

### 4.5. Dose- and time-dependent modulation of genistein’s neuroprotective mechanisms

General observations without qualitative analysis in the current study indicate that the neuroprotective pathways of genistein, as antioxidative, anti-inflammatory, and anti-apoptotic, are responsive to dose and time-dependent effects.

At low doses (≤5 mg/kg), genistein enhanced its antioxidant defense effect, primarily by stimulating Nrf2. Intermediate doses (5−15 mg/kg) showed an extended effect, with pathways such as the inhibition of NF-κB, NLRP3, the activation of PI3K/Akt-mTOR, and GLP-1 signaling, as well as an increase in the level of Bcl-2, demonstrating both anti-inflammatory and anti-apoptotic effects. Although only a few studies have performed higher doses (>15 mg/kg), additional signaling routes, including JAK/STAT3 and Wnt/Ca2 + , were found to be activated by stronger stimulation, suggesting that more sophisticated cell survival pathways are engaged by more robust stimulation.

In terms of the timing of treatment. earlier treatment before the insult was associated with more significant changes in infarct size and more effective inhibition of oxidative, inflammatory, and apoptotic molecular products. Long-term treatment (> 7 days) promoted the remodelling of neuroglia, such as the switching of astrocyte phenotype [33], suggesting additional benefits in the course of post-stroke recovery.

In line with *in vivo* evidence, *in vitro* experiments also indicated concentration and time-based effects. An increase in micromolar concentrations (or 50 µM and above) and exposure times (6–24 hours) resulted in more pronounced oxidative stress and modulation of the apoptotic pathway, further confirming the dose-responsive effect of genistein in experimental models.

In general, these results highlight the importance of optimizing the dosage and timing of genistein delivery to achieve the maximum neuroprotective effects and molecular stability of genistein at various time points following ischemic injury.

### 4.6. Limitations

Some of the limitations in this systematic review are that many studies mainly rely on preclinical models, which have focused on animal-based *in vivo* experiments. These models provide information regarding the neuroprotective mechanisms of genistein, but they may not accurately replicate the complex pathophysiology of ischemic stroke in humans. This restricts the immediate applicability of the results in clinical practice. Another limitation is the variation in dosage and administration routes between *in vitro* and *in vivo studies*. This complicates the standardization of the therapeutic protocols of genistein. In addition, various studies have varied in the selection of the ischemic model, which can result in varying findings on the effectiveness of genistein when used in different experiments. Also, there is no existing literature on long-term effects, so the long-term effects and possible side effects of genistein are not fully understood. These restrictions indicate that additional clinical studies and standard practices are required to confirm the therapeutic efficacy of genistein on ischemic stroke in humans. Ultimately, numerous studies have focused on molecular pathways, potentially overlooking other critical mechanisms involved in ischemic injury and recovery. Future research addressing these limitations is significant for advancing genistein as a viable therapeutic option for stroke patients.

## 5. Conclusion

Genistein has shown definite promise as a neuroprotective against stroke. Comparing the *in vitro* and *in vivo* researchers, genistein has a profound impact via anti-inflammatory, anti-oxidative, and anti-apoptotic pathways. Based on the results of this review, genistein is able to modulate several molecular targets, including Nrf2, NF-κB, and NLRP3, which play a major role in alleviating neuronal injury induced by ischemic stroke. Considerable *in vitro* and *in vivo* studies have recognized numerous therapeutic targets and pathways, suggesting that genistein can be effective at various dosages. To further justify the potential inclusion of genistein in clinical trials, additional research is needed on its effects on various strains *in vivo* and on different types of cells *in vitro*. Such future studies would further develop the analysis of the existing systematic literature review by incorporating additional data, thereby providing further evidence for the use of genistein in clinical practice.

## Supporting information

S1 Table Inter-rater reliability between reviewers. Additional details on observed agreement, expected agreement, and Cohen’s kappa coefficient are provided in the file.
(DOCX)

S1 File PRISMA 2020 Checklist. (DOCX)

## References

[1] Miao Z-Y, Xia X, Che L, Song Y-T. Genistein attenuates brain damage induced by transient cerebral ischemia through up-regulation of Nrf2 expression in ovariectomized rats. Neurol Res. 2018;40(8):689–95. doi: 10.1080/01616412.2018.1462879 29688134

[2] Andersson J, Rejnö Å, Jakobsson S, Hansson P-O, Nielsen SJ, Björck L. Symptoms at stroke onset as described by patients: a qualitative study. BMC Neurol. 2024;24(1):150. doi: 10.1186/s12883-024-03658-4 38702612 PMC11067237

[3] Lu L-Y, Liu Y, Gong Y-F, Zheng X-Y. A preliminary report: genistein attenuates cerebral ischemia injury in ovariectomized rats via regulation of the PI3K-Akt-mTOR pathway. Gen Physiol Biophys. 2019;38(5):389–97. doi: 10.4149/gpb_2019024 31595881

[4] Xie J, Li X, Zhang L, Liu C, Leung JW-H, Liu P, et al. Genistein-3’-sodium sulfonate ameliorates cerebral ischemia injuries by blocking neuroinflammation through the α7nAChR-JAK2/STAT3 signaling pathway in rats. Phytomedicine. 2021;93:153745. doi: 10.1016/j.phymed.2021.153745 34634743

[5] Cortina B, Torregrosa G, Castelló-Ruiz M, Burguete MC, Moscardó A, Latorre A, et al. Improvement of the circulatory function partially accounts for the neuroprotective action of the phytoestrogen genistein in experimental ischemic stroke. Eur J Pharmacol. 2013;708(1–3):88–94. doi: 10.1016/j.ejphar.2013.02.016 23461855

[6] Qian Y, Guan T, Huang M, Cao L, Li Y, Cheng H, et al. Neuroprotection by the soy isoflavone, genistein, via inhibition of mitochondria-dependent apoptosis pathways and reactive oxygen induced-NF-κB activation in a cerebral ischemia mouse model. Neurochem Int. 2012;60(8):759–67. doi: 10.1016/j.neuint.2012.03.011 22490611

[7] Liu C, Liu S, Xiong L, Zhang L, Li X, Cao X, et al. Genistein-3’-sodium sulfonate Attenuates Neuroinflammation in Stroke Rats by Down-Regulating Microglial M1 Polarization through α7nAChR-NF-κB Signaling Pathway. Int J Biol Sci. 2021;17(4):1088–100. doi: 10.7150/ijbs.56800 33867831 PMC8040300

[8] Wang S, Wang J, Wei H, Gu T, Wang J, Wu Z, et al. Genistein Attenuates Acute Cerebral Ischemic Damage by Inhibiting the NLRP3 Inflammasome in Reproductively Senescent Mice. Front Aging Neurosci. 2020;12:153. doi: 10.3389/fnagi.2020.00153 32625078 PMC7311792

[9] Qian Y, Cao L, Guan T, Chen L, Xin H, Li Y, et al. Protection by genistein on cortical neurons against oxidative stress injury via inhibition of NF-kappaB, JNK and ERK signaling pathway. Pharm Biol. 2015;53(8):1124–32. doi: 10.3109/13880209.2014.962057 25715966

[10] Page MJ, McKenzie JE, Bossuyt PM, Boutron I, Hoffmann TC, Mulrow CD, et al. The PRISMA 2020 statement: an updated guideline for reporting systematic reviews. BMJ. 2021;372:n71. doi: 10.1136/bmj.n71 33782057 PMC8005924

[11] Law M, Stewart D, Pollock N, Letts L, Bosch J, Westmoreland M. Critical review form: quantitative studies. Hamilton (ON): McMaster University Evidence-based Practice Research Group; 1998.

[12] Wolden M, Hill B, Voorhees S. Predicting Success for Student Physical Therapists on the National Physical Therapy Examination: Systematic Review and Meta-Analysis. Phys Ther. 2020;100(1):73–89. doi: 10.1093/ptj/pzz145 31584670

[13] Trieu VN, Uckun FM. Genistein is neuroprotective in murine models of familial amyotrophic lateral sclerosis and stroke. Biochem Biophys Res Commun. 1999;258(3):685–8. doi: 10.1006/bbrc.1999.0577 10329446

[14] Li H-C, Zhang G-Y. Inhibitory effect of genistein on activation of STAT3 induced by brain ischemia/reperfusion in rat hippocampus. Acta Pharmacol Sin. 2003;24(11):1131–6. 14627498

[15] Liang H-W, Qiu S-F, Shen J, Sun L-N, Wang J-Y, Bruce IC, et al. Genistein attenuates oxidative stress and neuronal damage following transient global cerebral ischemia in rat hippocampus. Neurosci Lett. 2008;438(1):116–20. doi: 10.1016/j.neulet.2008.04.058 18467029

[16] Schreihofer DA, Redmond L. Soy phytoestrogens are neuroprotective against stroke-like injury in vitro. Neuroscience. 2009;158(2):602–9. doi: 10.1016/j.neuroscience.2008.10.003 18976694 PMC2652887

[17] Ma Y, Sullivan JC, Schreihofer DA. Dietary genistein and equol (4’, 7 isoflavandiol) reduce oxidative stress and protect rats against focal cerebral ischemia. Am J Physiol Regul Integr Comp Physiol. 2010;299(3):R871-7. doi: 10.1152/ajpregu.00031.2010 20631292

[18] Castelló-Ruiz M, Torregrosa G, Burguete MC, Salom JB, Gil JV, Miranda FJ, et al. Soy-derived phytoestrogens as preventive and acute neuroprotectors in experimental ischemic stroke: influence of rat strain. Phytomedicine. 2011;18(6):513–5. doi: 10.1016/j.phymed.2011.02.001 21420287

[19] Wang R, Tu J, Zhang Q, Zhang X, Zhu Y, Ma W, et al. Genistein attenuates ischemic oxidative damage and behavioral deficits via eNOS/Nrf2/HO-1 signaling. Hippocampus. 2013;23(7):634–47. doi: 10.1002/hipo.22126 23536494

[20] Shambayati M, Patel M, Ma Y, Cunningham RL, Schreihofer DA. Central inflammatory response to experimental stroke is inhibited by a neuroprotective dose of dietary soy. Brain Res. 2014;1593:76–82. doi: 10.1016/j.brainres.2014.09.042 25261694 PMC12885063

[21] Shi R, Wang S, Qi X, Chen S, Chen P, Zhang Q. Lose dose genistein inhibits glucocorticoid receptor and ischemic brain injury in female rats. Neurochemistry Int. 2014;65:14–22. 10.1016/j.neuint.2013.12.002 24334056

[22] Wang S, Wei H, Cai M, Lu Y, Hou W, Yang Q, et al. Genistein attenuates brain damage induced by transient cerebral ischemia through up-regulation of ERK activity in ovariectomized mice. Int J Biol Sci. 2014;10(4):457. 24719563 10.7150/ijbs.7562 PMC3979998

[23] Aras AB, Guven M, Akman T, Alacam H, Kalkan Y, Silan C, et al. Genistein exerts neuroprotective effect on focal cerebral ischemia injury in rats. Inflammation. 2015;38(3):1311–21. doi: 10.1007/s10753-014-0102-0 25567369

[24] Banecka-Majkutewicz Z, Kadziński L, Grabowski M, Bloch S, Kaźmierkiewicz R, Jakóbkiewicz-Banecka J, et al. Evidence for interactions between homocysteine and genistein: insights into stroke risk and potential treatment. Metab Brain Dis. 2017;32(6):1855–60. doi: 10.1007/s11011-017-0078-1 28748495

[25] Li L, Xue J, Liu R, Li X, Lai L, Xie J, et al. Neuroprotective effects of genistein-3’-sodium sulfonate on focal cerebral ischemia in rats. Neurosci Lett. 2017;646:43–8. doi: 10.1016/j.neulet.2017.02.046 28237799

[26] Morán J, Perez-Basterrechea M, Garrido P, Díaz E, Alonso A, Otero J, et al. Effects of Estrogen and Phytoestrogen Treatment on an In Vitro Model of Recurrent Stroke on HT22 Neuronal Cell Line. Cell Mol Neurobiol. 2017;37(3):405–16. doi: 10.1007/s10571-016-0372-1 27059741 PMC11482143

[27] Rajput MS, Sarkar PD, Nirmal NP. Inhibition of DPP-4 Activity and Neuronal Atrophy with Genistein Attenuates Neurological Deficits Induced by Transient Global Cerebral Ischemia and Reperfusion in Streptozotocin-Induced Diabetic Mice. Inflammation. 2017;40(2):623–35. doi: 10.1007/s10753-017-0509-5 28091829

[28] Wang Y-X, Tian K, He C-C, Ma X-L, Zhang F, Wang H-G, et al. Genistein inhibits hypoxia, ischemic-induced death, and apoptosis in PC12 cells. Environ Toxicol Pharmacol. 2017;50:227–33. doi: 10.1016/j.etap.2017.01.022 28192752

[29] Xue J, Li X, Xie J, Liu R, Huang C, Huang Z. Neuroprotective effects of genistein-3’-sodium sulfonate in a rat middle cerebral artery occlusion model: roles of enhancing antioxidant ability and regulating NO/NOS system. Int J Clin Exp Med. 2018;11(11):12326–32.

[30] Li Y, Zhang J-J, Chen R-J, Chen L, Chen S, Yang X-F, et al. Genistein mitigates oxidative stress and inflammation by regulating Nrf2/HO-1 and NF-κB signaling pathways in hypoxic-ischemic brain damage in neonatal mice. Ann Transl Med. 2022;10(2):32. doi: 10.21037/atm-21-4958 35282070 PMC8848430

[31] Wang S, Zhang Z, Wang J, Ma L, Zhao J, Wang J, et al. Neuronal GPER Participates in Genistein-Mediated Neuroprotection in Ischemic Stroke by Inhibiting NLRP3 Inflammasome Activation in Ovariectomized Female Mice. Mol Neurobiol. 2022;59(8):5024–40. doi: 10.1007/s12035-022-02894-4 35661323

[32] Xie T, Shuang L, Liu G, Zhao S, Yuan Z, Cai H, et al. Insight into the Neuroprotective Effect of Genistein-3’-Sodium Sulfonate Against Neonatal Hypoxic-Ischaemic Brain Injury in Rats by Bioinformatics. Mol Neurobiol. 2023;60(2):807–19. doi: 10.1007/s12035-022-03123-8 36370154 PMC9849302

[33] Liu R, Yu Y, Ge Q, Feng R, Zhong G, Luo L, et al. Genistein-3’-sodium sulfonate promotes brain functional rehabilitation in ischemic stroke rats by regulating astrocytes polarization through NF-κB signaling pathway. Chem Biol Interact. 2024;400:111159. doi: 10.1016/j.cbi.2024.111159 39059603

[34] Li L, Liu S, Wang M, Li M, Liu Y, Chen H, et al. Gen inhibiting the Wnt/Ca2+ signaling pathway alleviates cerebral ischemia/reperfusion injury. Sci Rep. 2025;15(1):4661. doi: 10.1038/s41598-025-88136-8 39920331 PMC11805899

[35] Yu Y, Liao X, Xing K, Xie Z, Xie N, He Y, et al. Genistein-3’-sodium sulfonate suppresses NLRP3-mediated cell pyroptosis after cerebral ischemia. Metab Brain Dis. 2025;40(1):99. doi: 10.1007/s11011-025-01530-z 39808354

[36] Chen J, Nagayama T, Jin K, Stetler RA, Zhu RL, Graham SH, et al. Induction of caspase-3-like protease may mediate delayed neuronal death in the hippocampus after transient cerebral ischemia. J Neurosci. 1998;18(13):4914–28. doi: 10.1523/JNEUROSCI.18-13-04914.1998 9634557 PMC6792571

[37] Shah ZA, Li R, Ahmad AS, Kensler TW, Yamamoto M, Biswal S, et al. The flavanol (-)-epicatechin prevents stroke damage through the Nrf2/HO1 pathway. J Cereb Blood Flow Metab. 2010;30(12):1951–61. doi: 10.1038/jcbfm.2010.53 20442725 PMC3002885

[38] Noshita N, Lewén A, Sugawara T, Chan PH. Akt phosphorylation and neuronal survival after traumatic brain injury in mice. Neurobiol Dis. 2002;9(3):294–304. doi: 10.1006/nbdi.2002.0482 11950275

[39] Chi X-X, Zhang T, Chu X-L, Zhen J-L, Zhang D-J. The regulatory effect of Genistein on granulosa cell in ovary of rat with PCOS through Bcl-2 and Bax signaling pathways. J Vet Med Sci. 2018;80(8):1348–55. doi: 10.1292/jvms.17-0001 29937456 PMC6115251

[40] Hu WS, Lin YM, Ho TJ, Chen RJ, Li YH, Tsai FJ, et al. Genistein suppresses the isoproterenol-treated H9c2 cardiomyoblast cell apoptosis associated with P-38, erk1/2, JNK, and NF κ B signaling protein activation. Am J Chinese Med. 2013;41(05):1125–36. 10.1142/S0192415X13500766 24117073

[41] Si H, Liu D. Isoflavone genistein protects human vascular endothelial cells against tumor necrosis factor-alpha-induced apoptosis through the p38beta mitogen-activated protein kinase. Apoptosis. 2009;14(1):66–76. doi: 10.1007/s10495-008-0283-9 19082897 PMC2729690

[42] Ji G, Yang Q, Hao J, Guo L, Chen X, Hu J, et al. Anti-inflammatory effect of genistein on non-alcoholic steatohepatitis rats induced by high fat diet and its potential mechanisms. Int Immunopharmacol. 2011;11(6):762–8. doi: 10.1016/j.intimp.2011.01.036 21320636

[43] Kang JL, Lee HW, Lee HS, Pack IS, Chong Y, Castranova V, et al. Genistein prevents nuclear factor-kappa B activation and acute lung injury induced by lipopolysaccharide. Am J Respir Crit Care Med. 2001;164(12):2206–12. doi: 10.1164/ajrccm.164.12.2104017 11751189

[44] Chen Y, Le TH, Du Q, Zhao Z, Liu Y, Zou J, et al. Genistein protects against DSS-induced colitis by inhibiting NLRP3 inflammasome via TGR5-cAMP signaling. Int Immunopharmacol. 2019;71:144–54. doi: 10.1016/j.intimp.2019.01.021 30901677

[45] Li Z, Li Y, Zhao J, Pang Z, Guo F. Genistein inhibits Nlrp3/caspase-1 signalling to alleviate traumatic brain injury-induced anxiety-like behaviours in rats. Acta Neuropsychiatr. 2024;36(4):242–8. doi: 10.1017/neu.2024.22 39327861

[46] Zhang L-Y, Xue H-G, Chen J-Y, Chai W, Ni M. Genistein induces adipogenic differentiation in human bone marrow mesenchymal stem cells and suppresses their osteogenic potential by upregulating PPARγ. Exp Ther Med. 2016;11(5):1853–8. doi: 10.3892/etm.2016.3120 27168816 PMC4840518

[47] Babu PVA, Si H, Fu Z, Zhen W, Liu D. Genistein prevents hyperglycemia-induced monocyte adhesion to human aortic endothelial cells through preservation of the cAMP signaling pathway and ameliorates vascular inflammation in obese diabetic mice. J Nutr. 2012;142(4):724–30. doi: 10.3945/jn.111.152322 22399524 PMC3301991

[48] Ding Q, Pi A, Hao L, Xu T, Zhu Q, Shu L, et al. Genistein Protects against Acetaldehyde-Induced Oxidative Stress and Hepatocyte Injury in Chronic Alcohol-Fed Mice. J Agric Food Chem. 2023;71(4):1930–43. doi: 10.1021/acs.jafc.2c05747 36653166

[49] Kim M-H, Kang K-S, Lee Y-S. The inhibitory effect of genistein on hepatic steatosis is linked to visceral adipocyte metabolism in mice with diet-induced non-alcoholic fatty liver disease. Br J Nutr. 2010;104(9):1333–42. doi: 10.1017/S0007114510002266 20687969

[50] Gupta SK, Dongare S, Mathur R, Mohanty IR, Srivastava S, Mathur S, et al. Genistein ameliorates cardiac inflammation and oxidative stress in streptozotocin-induced diabetic cardiomyopathy in rats. Mol Cell Biochem. 2015;408(1–2):63–72. doi: 10.1007/s11010-015-2483-2 26092427

[51] Wang L, Li A, Liu Y, Zhan S, Zhong L, Du Y, et al. Genistein protects against acetaminophen-induced liver toxicity through augmentation of SIRT1 with induction of Nrf2 signalling. Biochem Biophys Res Commun. 2020;527(1):90–7. doi: 10.1016/j.bbrc.2020.04.100 32446397

