# Supplementary material for: Therapeutic mechanisms of genistein in ischemic stroke: A systematic review of in vivo and in vitro studies
Source: PLoS One. 2025 Dec 23;20(12):e0338590. doi: 10.1371/journal.pone.0338590 (PMC12725566; doi:10.1371/journal.pone.0338590)
Supplement: S1 Table — Additional details on observed agreement, expected agreement, and Cohen’s kappa coefficient are provided in the file. (DOCX) [file pone.0338590.s001.docx]

**Table S1. Inter-rater Reliability Between Reviewers**

|  | Reviewer 2: Relevant | Reviewer 2: Not Relevant | Total |
| --- | --- | --- | --- |
| Reviewer 1: Relevant | 262 | 3 | 265 |
| Reviewer 1: Not Relevant | 16 | 60 | 76 |
| Total | 278 | 63 | 341 |

- **Observed Agreement (Po)**: 0.944 (94.4%)
- **Expected Agreement (Pe)**: 0.675 (67.5%)
- **Cohen's Kappa (κ)**: 0.829

According to the interpretation scale, a Kappa value of 0.83 indicates almost perfect agreement between the two reviewers.
